# Modulation of autophagy, apoptosis and oxidative stress: a clue for repurposing metformin in photoaging

**DOI:** 10.1007/s10787-022-01041-8

**Published:** 2022-08-01

**Authors:** Dalia Kamal Mostafa, Omnia A. Nayel, Shaymaa Abdulmalek, Ahmed A. Abdelbary, Cherine A. Ismail

**Affiliations:** 1 Department of Clinical Pharmacology, Faculty of Medicine, Alexandria University, Al-Mouassat medical Campus, Elhadara, Alexandria, Postal code: 21561 Egypt; 2 Biochemistry Department, Faculty of Science, Alexandria University, Alexandria, Egypt; 3 Centre of Excellence for Preclinical Study, Pharmaceutical and Fermentation Industries Development Centre, City of Scientific Research and Technologies Application, Alexandria, Egypt; 4 Department of Dermatology, Venereology and Andrology, Faculty of Medicine, Alexandria University, Alexandria, Egypt

**Keywords:** Caspase, Cathepsin D, Coenzyme Q10, Ultraviolet irradiation, Nrf-2

## Abstract

Long-term sun exposure is the commonest cause of photoaging, where mutual interplay between autophagy, oxidative stress, and apoptosis is incriminated. In combating photoaging, pharmacological approaches targeted to modulate autophagy are currently gaining more ground. This study aimed to examine repurposing metformin use in such context with or without the antioxidant coenzyme Q10 (coQ10) in ultraviolet A (UVA) irradiation-induced skin damage. The study was conducted on 70 female CD1 mice that were randomly assigned into seven groups (10/group): normal control, vehicle-treated-UVA-exposed mice, three metformin UVA-exposed groups (Topical 1 and 10%, and oral 300 mg/kg), topical coQ10 (1%)-treated mice, and combined oral metformin with topical coQ10-treated UVA-exposed mice. After UVA-exposure for 10 weeks (3 times/week), macroscopic signs of photoaging were evaluated. Mice were then euthanized, and the skin was harvested for biochemical estimation of markers for oxidative stress, inflammation, matrix breakdown, and lysosomal function. Histopathological signs of photoaging were also evaluated with immunohistochemical detection of associated changes in autophagic and apoptotic markers. Metformin, mainly by topical application, improved clinical and histologic signs of photoaging. This was associated with suppression of the elevated oxidative stress, IL-6, matrix metalloproteinase 1, and caspase, with induction of cathepsin D and subsequent change in anti-LC3 and P62 staining in skin tissue. In addition to metformin antioxidant, anti-inflammatory, and antiapoptotic activities, its anti-photoaging effect is mainly attributed to enhancing autophagic flux by inducing cathepsin D. Its protective effect is boosted by coQ10, which supports their potential use in photoaging.

## Introduction

Autophagy is a cellular degradation process, through which the cell gets rid of defective, aged, or damaged organelles and macromolecules. This evolutionary conserved process is crucial in maintaining cellular homeostasis against environmental stresses. Since dysregulated autophagy is linked to many human disorders, pharmacological activation or inhibition of autophagy is gaining ground in experimental and clinical research (Anding and Baehrecke 2017; Glick et al. 2010).

In mammals, the skin acts as the front-line barrier against exogenous insults (Chen et al. 2012). Unlike chronological aging, which is predetermined by individual’s physiological predisposition, chronic exposure to ultraviolet (UV) radiation is the major cause of skin damage in photoaging. UVA radiation induces reactive oxygen species (ROS) in epidermal and dermal cells that exceed their clearance capacity. The damaged molecules and organelles, whose turnover is controlled by autophagy, accumulate, and trigger a series of aging-related signaling pathways, promoting cellular senescence. The degree of sun exposure, particularly the UVA radiation is, thus, the most important determinant or causal root of photoaging (Pandel et al. 2013).

Previous studies reported autophagy activation by the UV radiation-induced damage (Sample and He 2017). Conversely, other studies revealed autophagic suppression, suggesting that autophagy may be a denominator for extrinsic skin aging (Remenapp et al. 2017). Moreover, inhibiting autophagy under conditions of UV-radiation exposure reduced cell survival and enhanced apoptosis *in-vitro* (Chen et al. 2012). These findings raise interest in pharmacological activation of autophagy as a novel approach to cytoprotection against photoaging.

Given the recently uncovered role of autophagy in keratinocytes and dermal fibroblasts cytopathobiology (Chen et al. 2016), many pharmacological and nutritional agents were investigated for their potential to modulate skin autophagy (Chen et al. 2016; Vitale et al. 2013). The antidiabetic drug, metformin is a strong autophagy inducer (Li et al. 2014). However, its effect on skin autophagy was not addressed before. Though metformin use is gaining interest for treating dermatological disorders (Bubna 2016) and is under a breakthrough study to be validated as the first-ever anti-aging medication (Klimova et al. 2018), its impact on photoaging is only recently getting to be explored. Its benefit was mainly attributed to favourably modifying the redox state (Cui et al. 2019).

We aimed to evaluate metformin effects in photoaging in view of its role in autophagic regulation. The close association of photoaging and oxidative stress is uncontested. The UV-induced damage was reported to be prevented by antioxidant treatment (Pandel et al. 2013). Since ROS are both inducer and target for autophagy, it was of interest to study the effect of inducing autophagy by metformin versus the mono- and combined-therapy with the well-known antioxidant; coenzyme Q10 (coQ10) in UVA-induced photoaged mice. We previously demonstrated a protective effect of the oral coQ10 against photoaging with a differential modulation of autophagy in various skin cells (Mostafa et al. 2020). Herein, we explored whether these results are reproduced by topical coQ10 application. We also aimed to analyse the role of autophagy/apoptosis crosstalk in the proposed effects of the studied drugs against the UV-induced skin damage.

## Materials and methods

### Animals

A total of 70 female CD1 mice (25–35 g) were purchased from the animal house of the Faculty of Medicine, Alexandria University and were maintained under standard laboratory conditions with free access to food and water. A one-week acclimatization period was allowed before starting the experiment. All effort was made to minimize animal suffering, and animal care was according to the NIH Guide for Care and Use of Laboratory Animals. The study complies with the ARRIVE guidelines and experiments were conducted after approval of the local Ethics Committee of the Faculty of Medicine, Alexandria University. (June 2018: 0,305,121).

### Drugs and chemicals

The following drugs and chemicals were used in the study: Metformin (Glucophage^®^, Bristol-Myers Squibb), coenzyme Q10^®^ (Co-Enzyme Q10, Mepaco-Medifood, Egypt), Polypropylene glycol (Elgomhoria, Egypt), and Malondialdehyde (MDA) and reduced glutathione (GSH) detection assay Kits (Abcam, #ab118970 and #ab239727, respectively). Also, rat Interleukin-6 (IL-6) ELISA kit (Biocompare), rat matrix metalloproteinase-1 (MMP-1) ELISA kit (MyBiosource, (Cat# MBS764916), nuclear factor erythroid-related factor 2 (Nrf-2) antibody (cell signaling-20733) caspase 3 antibody (Lab Vision Corporation, Neo Markers, Fremont, USA, RB1197R7), microtubule associated protein 1 light chain 3 alpha (LC3-II) mouse monoclonal antibody and rabbit polyclonal antibody to sequestosome 1, known as anti P62 antibody (Gene Tex, international corporation, USA, GTX82986, GTX100685, respectively), immunostaining detection system (Lab Vision Corporation Neo Markers, Fremont, USA, TP-015-HD), cathepsin D antibody (Abcam, #ab19555), and β-actin antibody (cell signalling Technology, 8457 s) were used. All other chemicals were analytical-grade commercial products.

### Experimental design

The effect of oral metformin either alone or in combination with topical coQ10 on photoaging was investigated, as well as that of the topically applied metformin in two different concentrations. Animals were randomly assigned into seven groups of 10 mice each, as following: normal control, vehicle-treated-UVA-exposed mice, three metformin-treated UVA-exposed groups (Topical metformin 1% (Rao et al. 2013), and 10% (Araoye et al. 2020), and oral metformin 300 mg/kg /day (Wu et al. 2013), topical coQ10 (1%)-treated UVA-exposed mice (Davis et al. 2017), and finally combined oral metformin with topical coQ10-treated UVA-exposed mice. Topical drug formulations were prepared in the aforementioned weight: volume percentage solutions in a standard dermatologic vehicle of 70% ethanol and 30% polypropylene glycol. A total volume of 400 µl of the topical preparations were applied to the shaved skin area of each treated mouse (Lehraiki et al. 2014). Oral metformin is given in 2% gum acacia as a vehicle. In each group, animals were randomly divided into 2 cages (5 mice/cage) to ensure the equal exposure to UVA for induction of photoaging.

### Experimental procedures

Before starting the experiment, an area of about 2.5 × 3 cm from the dorsal skin of each mouse was shaved clean. Except for the normal control mice, photoaging was induced by exposure of all animals to UVA using UVA lamp (Waldmann UV800, Germany) that exclusively emits UVA in the range of 320–400 nm (peak at 365 nm). For calibration, the irradiation intensity at 18 cm from the light source was preliminarily measured by a UV meter in the department of electrical engineering, Faculty of engineering, Alexandria University. The mice in each cage were scheduled to be exposed to UVA radiation 3 times a week for 10 weeks starting by one minimal erythema dose (MED) = 80 mj/cm^2^ in the first week, then escalated weekly by a one MED to reach 4 MEDs by the fourth week. Mice were then continued to be irradiated by 4 MEDs for the remaining 6 weeks till the end of the study. Normal control mice were exposed instead to white light emitted from normal fluorescent tubes for the whole duration (Kong et al. 2013; Lin et al. 2014; Zhan et al. 2016). Topical drugs or vehicle were applied once daily half an hour before UVA exposure, while oral metformin or its vehicle were administered twice daily by oral gavage for 10 weeks from the beginning of the study.

### Macroscopic evaluation of photoaging

Detection and follow up of the macroscopic signs of photodamage were conducted by weekly photographing the dorsal skin of each mouse using a Nikon D7000 digital camera with a macro 100 lens (Nikon, Tokyo, Japan). At the end of the study, a final evaluation of the signs of photodamage was conducted by examination of photos captured following visualization under dermatoscopic magnification (Dermlite4W, 3 Gen, Inc.). The degree of photodamage was assessed using a modified grading score ranging from 0 to 6 for normal skin to severely damaged skin, respectively (Kong et al. 2013). Estimation of skin elasticity was done by using the pinch test according to the method of Bryce and Bogdan (Bryce 1991). Briefly, the time (in seconds) of the dorsal skin recovery from stretching was recorded for 3 times under general anesthesia (ketamine, 80 mg/kg) (Levin-Arama et al. 2016) and the average value was calculated for each mouse. Animals were then euthanized, and their dorsal skin was harvested and divided into multiple parts for biochemical and histopathological examination.

### Biochemical estimates for oxidative stress, IL-6, and MMP-1

Part of the excised skin was washed with phosphate buffered saline solution before homogenization in 10 ml cold potassium phosphate buffer per gram tissue and centrifugation at 4000 r.p.m. for 15 min. The supernatant was then removed and stored at − 80 ℃ for further assay of the oxidative state. Malondialdehyde (MDA) and reduced glutathione (GSH) were measured as indices of lipid peroxidation and endogenous antioxidants, respectively, by a colorimetric technique using commercial kits according to the manufacturer’s instructions. IL-6 and MMP-1, as markers of inflammation and collagen breakdown, respectively, were assessed by enzyme linked immunosorbent assay (ELISA) according to the manufacturer’s instruction.

### Western-blot analysis for cathepsin D and Nrf-2

Form the homogenized skin, 50 µg protein lysates from each sample were mixed with 2X loading buffer (130 mM Tris–HCl, pH 8.0, 30% (v/v) Glycerol, 4.6% (w/v) SDS, 0.02% Bromophenol blue, 2% DTT), boiled for 5 min then cooled at 4 ℃. Samples were separated on 12% SDS-PAGE mini-gel (1.6 ml H2O, 1.5 M Tris pH 8.8, 1.3 ml, 30% Acrylamide acrylamide/bisacrylamide (29:1 mix in 100 ml) 2.0 ml, 10% SDS, 10% APS, TEMED 2 ml) and run at 120 V. Proteins were transferred to a nitrocellulose membrane at 22 V overnight at 4 ℃. The membrane was washed 3 times with Tris-buffered saline containing Tween 20 (TBST) (50 mM Tris, pH 7.5, 150 mM NaCl, 0.05% Tween-20) and then incubated in blocking buffer (TBST containing 5% non-fat dry milk) for 1 h at RT and then incubated overnight with primary antibodies for Cathepsin D, Nrf-2, or β-actin diluted with TBST and 5% bovine serum albumin (BSA). After 3 times wash with TBST, the membrane was incubated for 1 h at RT with secondary antibody. Membranes were again washed three times with TBST, and bands were detected by alkaline phosphatase solution, then bands were quantified using Image J quantification software (Burnette 1981).

### Histopathological and immunohistochemical evaluation

The harvested skin biopsy from each animal was fixed in 10% buffered formalin, then processed in alcohol and xylene. Then, for each case, 5 micron-thick sections were cut from the formalin-fixed, paraffin-embedded blocks and stained with conventional H&E stain and examined under light microscopy for determination of the epidermal and dermal changes in each case. Epidermal atrophy, dermal fibrosis, dermal inflammation, and dermal vascular proliferation were histopathological evaluated, and each was semi-quantitatively graded into three grades, mild (score 1), moderate (score 2), and severe (score 3), while no change is scored as 0. For immunohistochemical study, the following primary antibodies were used, anti-LC3 mouse monoclonal antibody, anti-P62, and anti-caspase 3 (clone (CPP32) Ab-4, rabbit polyclonal antibody. Expression of all antibodies was visualized using the stretavidin-biotin-immunoenzymatic antigen detection system, which was performed according to manufacturer's protocol. The 5 micron-thick sections mounted on positively charged coated slides (Polysine, Bio Optica, Milano, Spain), were deparaffinized in xylene, then rehydrated in descending grades of alcohol and finally brought to water. Slides were incubated in 3% hydrogen peroxide for ten minutes for blocking endogenous peroxidase activity. Sections were subjected to pretreatment for epitope retrieval using 10 mM citrate buffer solution (pH 6.0) for 15 min in a microwave oven at 60 °C. Ultra V block was applied for a maximum of 5 min to block non-specific background staining. The primary antibodies for LC3, P63, and caspase 3 were then applied at a concentration of 1:600, 1:600 and 1: 1000, respectively and incubated overnight at 4 ℃ in the humidity chamber. The Biotinylated Goat anti-Polyvalent antibody was then applied followed by Streptavidin Peroxidase, DAB Plus substrate, and DAB Plus Chromagen. Counter staining of the slides was then done using Harris hematoxylin. Slides were dehydrated in ascending grades of alcohol, and then cleared in xylene. Positive and negative controls were included in all runs. Immuno-stained sections were examined under low power examination (× 100) to detect positive cytoplasmic staining for the three markers. For LC3 and P62 antibodies, positive staining was defined as dot-like cytoplasmic brownish staining (puncta), while both homogenous cytoplasmic staining and nuclear staining were omitted (Schläfli et al. 2015). Higher power examination was then performed to detect the expressing cell type. For quantitative analysis, immune-stained sections were examined under low power examination for detection of the area of highest staining (hot spot) where three high power fields (HPFs) were photographed. Photos were then uploaded to the image analysis software (Image J bundled with Java 1.8.0_172), and the number of positively stained cells was determined. Staining density was expressed as the percentage of the positive cells relative to the total number of cells of the same type. Then, the mean percentage of positive cells in the three HPFs for each slide was calculated.

### Statistical analysis

Data were tested by Kolmogorov–Smirnov (K-S) test for normality. Normally distributed data were analysed by the one-way analysis of variance (ANOVA) followed by Tukey’s test for multiple comparison. KrusKal wallis test was used for testing ordinal data and multiple comparisons were done using Dunn’s test. Statistics were done using GraphPad Prism software (version 7.0). *P* values < 0.05 were considered significant.

## Results

### Effect of drug-treatment on photoaging macroscopic changes and skin elasticity

**Fig. 1:**
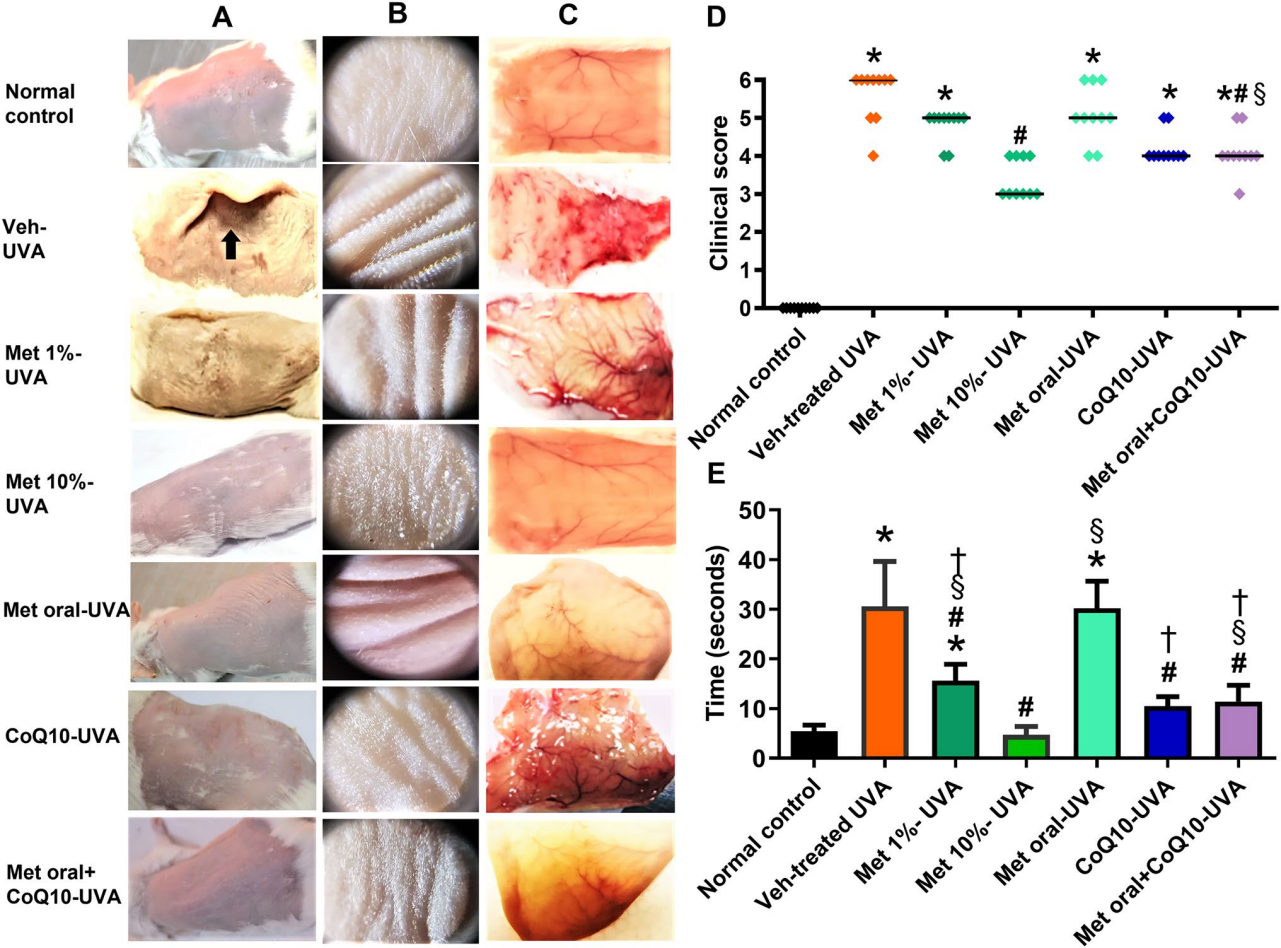
Clinical evaluation of macroscopic signs of photodamage and skin elasticity. Representative photographs of mice skin of the different groups are shown in (**A-C**), where set **A** represents the naked eye appearance of the mice skin and set **B** represents photos taken under the magnifications of the dermatoscope, while set **C** represents changes observed on the internal skin surface showing neovascularization, tissue oedema, and signs of inflammation. The deep wrinkles are illustrated in the skin of vehicle-treated UVA-exposed mice. Note the delayed skin relaxation after release from pinching as demarcated by the arrow. Attenuation of these wrinkles is observed with Met 10%, CoQ10- treatment, and the combined treated group. Statistical analysis of semi-quantitative scoring for the macroscopic signs of photoaging is presented by a scatter plot of individual values and medians (transverse lines) in (**D**), while results of assessment of pinch test time as a marker of skin elasticity is demonstrated as means ± SD in (**E**). * *P* < 0.05 versus normal control, **#** *P* < 0.05 versus veh-treated UVA group, § *P* < 0.05 versus Met 10%-UVA group, *† P* < 0.05 versus Met oral-UVA group. UVA ultraviolet A irradiation, Veh vehicle, Met, metformin, CoQ10 coenzyme Q10

Exposure to UVA was associated with development of the characteristic macroscopic signs of photoaging in the form of skin roughness, laxity, disappearance of fine striations, and the development of wrinkles that progressed to severe deep wrinkles in the vehicle-treated UVA- exposed mice at the end of the study. These changes were almost abolished in the Met 10%-treated mice being non significantly different from the normal control. Treatment with coQ10 when combined with oral metformin was associated with a significant amelioration of the macroscopic score of photoaging. (Fig. 1A-D) Likewise, testing of skin elasticity revealed a significant prolongation of the pinch test time in the vehicle-UVA-exposed group in comparison to normal control. A significant shortening of the recorded time was observed in the metformin 10%-treated mice and to a lesser extent in the coQ10 and the combined-treatment group versus the vehicle-treated UVA exposed mice. (Fig. 1E)

### Effect of drug-treatment on UVA-induced change in oxidative redox state

**Fig. 2:**
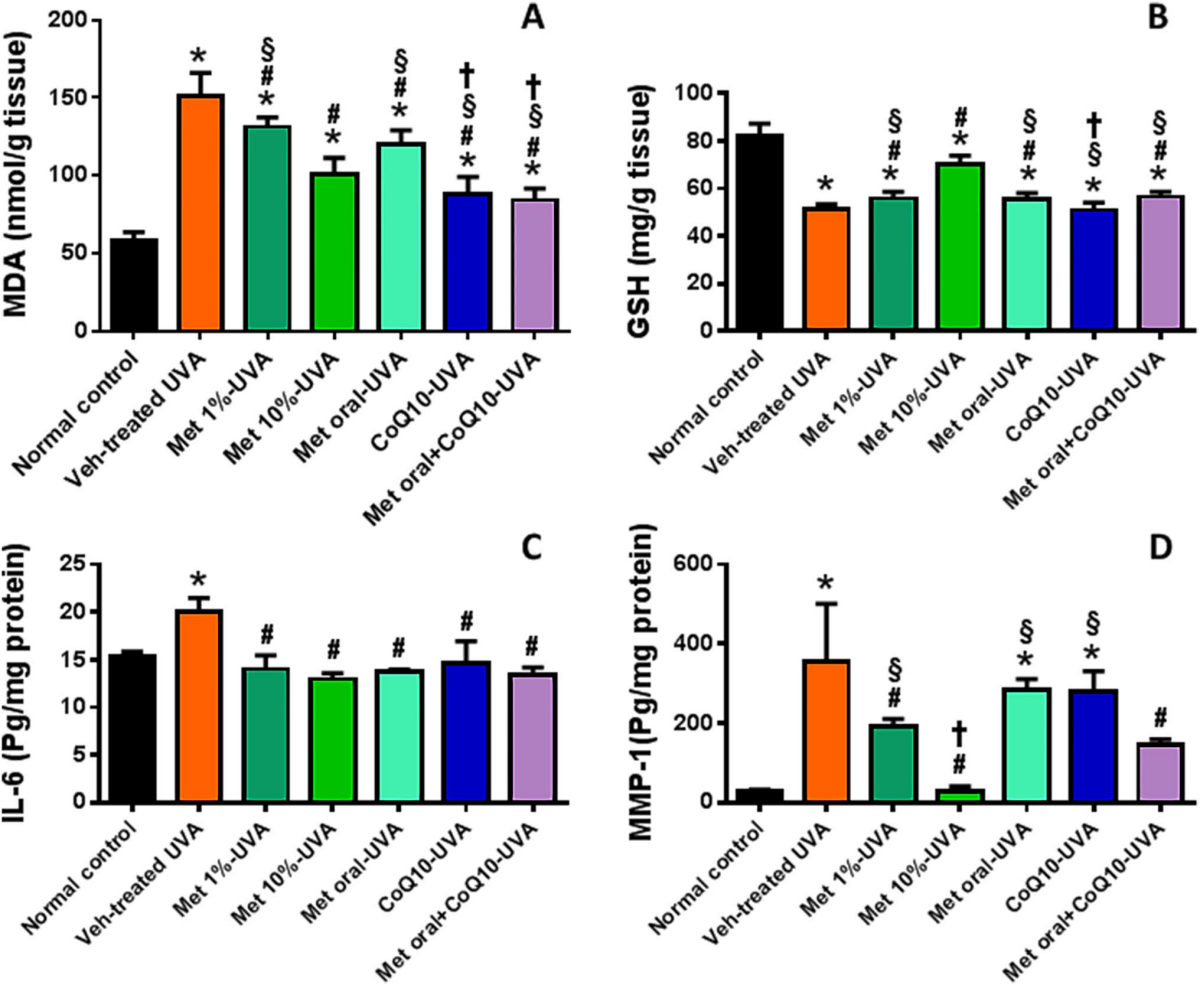
Effect of drug treatment on UVA-induced change in oxidative redox state, and IL-6 and MMP-1 in skin. Drug treatment reduced MDA in all mice groups (**A**), increased GSH except in coQ10-treated mice (**B**), normalized skin IL-6 (**C**), and variably inhibited skin MMP-1 (**D**). * *P* < 0.05 versus normal control, **#** *P* < 0.05 versus veh-treated UVA group, § *P* < 0.05 versus Met 10%-UVA group, †, *P* < 0.05 versus Met oral-UVA group. MDA malondialdehyde, GSH reduced glutathione, MMP-1 metalloproteinase-1, UVA ultraviolet A irradiation, Veh vehicle, Met metformin, CoQ10 coenzyme Q10

A significant increase in MDA as a marker of oxidative stress was detected in the skin of the UVA-exposed mice versus normal control. Topical or oral drug administration was associated with a significant reduction in MDA levels to varying degrees with the greater effect observed in the CoQ10-treated groups followed by metformin 10% treatment. An opposite trend was observed for GSH, whose level was significantly reduced in all UVA-exposed groups versus normal control. All drug treatments were associated with a significant inhibition of the UVA-induced-GSH reduction, except for the CoQ10-treated mice. (Fig. 2A, B).

### Effect of drug-treatment on UVA-induced change in IL-6 and MMP-1

For IL-6, UVA-exposure was associated with a significant increase in skin IL-6 versus normal control mice. Such increase was shown to be normalized in all drug treatment groups. Likewise, MMP-1 was markedly increased in the skin of vehicle-treated UVA-exposed mice, and this increase was significantly inhibited by variable levels in all drug-treated groups approaching almost normal levels in the metformin 10% treated mice. (Fig. 2C, D).

### Effect of drug treatment on UVA-induced change in cathepsin D and Nrf-2 expression

**Fig. 3:**
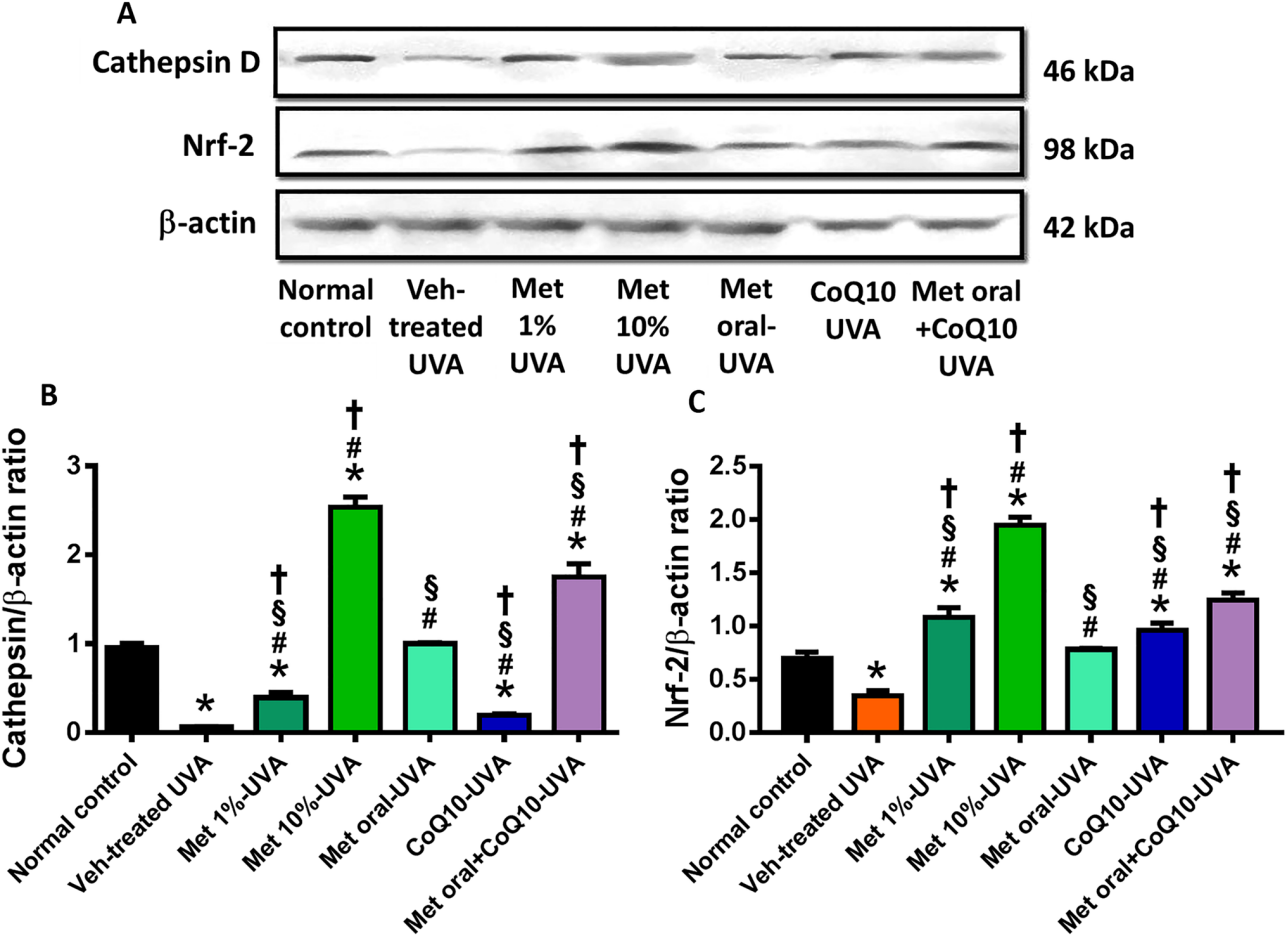
Representative immunoblots of skin cathepsin D and Nrf-2 with their quantitative evaluation. Total proteins were analysed using antibodies against cathepsin D or Nrf-2. β-actin was used as control for protein loading (**A**). Quantification of both proteins by image J are plotted as a ratio to β-actin in (**B**) and (**C**), respectively. * *P* < 0.05 versus normal control; # *P* < 0.05 versus veh-treated UVA group, § *P* < 0.05 versus Met 10%-UVA group, **†** *P* < 0.05 versus Met oral-UVA group. UVA ultraviolet A irradiation, Veh vehicle, Met metformin, CoQ10 coenzyme Q10

Western blot assessment of skin cathepsin expression, the important protease that mediates auto-lysosomal degradation during autophagy, revealed a significant inhibition of cathepsin D expression by UVA-exposure. Treatment with metformin was associated with a significant increase in cathepsin D expression with the highest values shown with the topical metformin 10%. Subtle but significant increase change was observed in the CoQ10-treated group. Skin Nrf-2 expression was reduced by UVA exposure versus normal control. Treatment with metformin and coQ10 significantly increased Nrf-2 expression, being highly expressed with topical metformin 10%. (Fig. 3).

### Effect of drug-treatment on UVA-induced histopathological changes

**Fig. 4:**
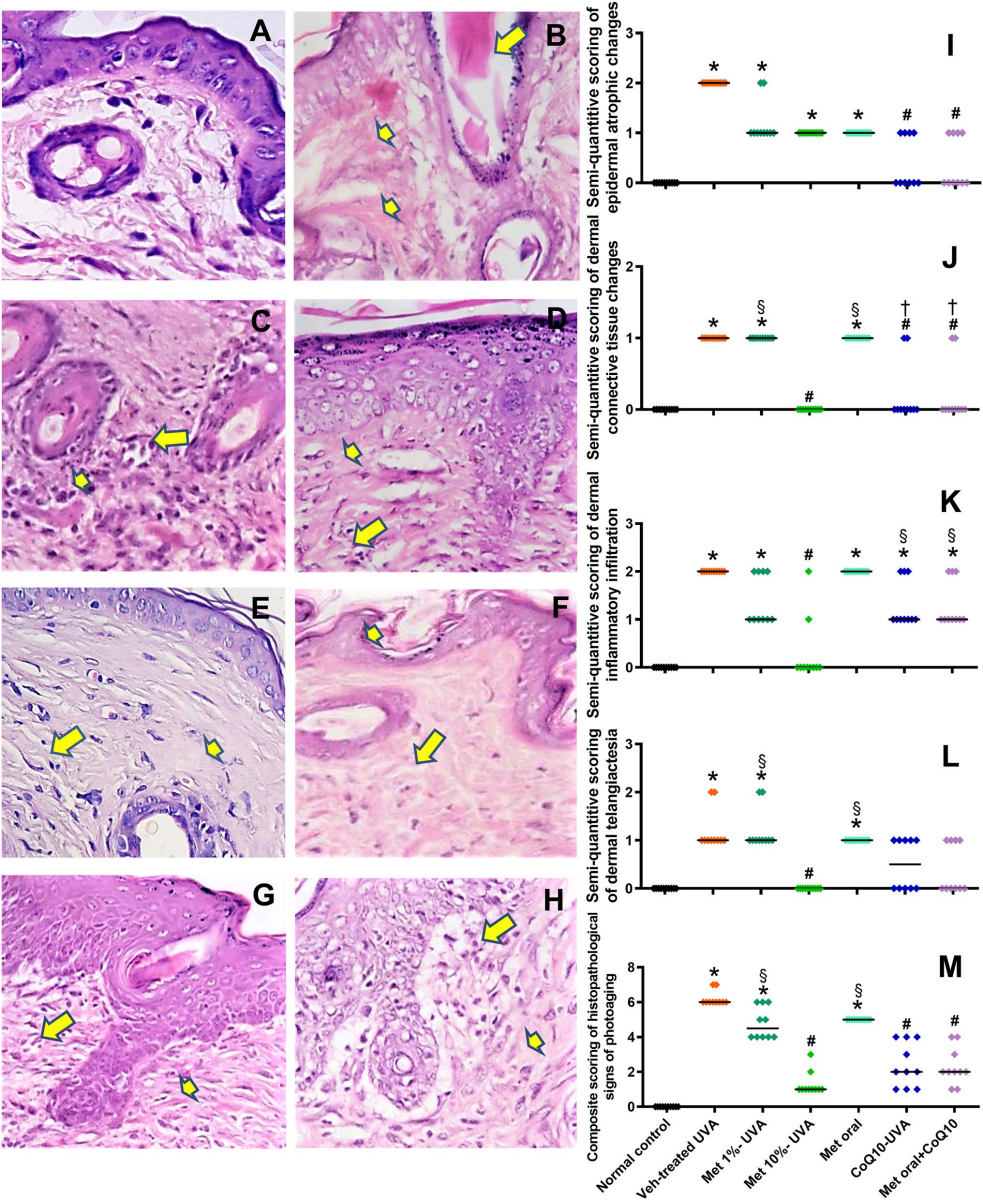
Effect of the treating drugs on the microscopic signs of photoaging. Representative photomicrographs of light microscopic picture of H&E-stained skin tissue sections (Mic. Mag. × 200), showing skin of normal control mouse with unremarkable epidermis and dermis and few scattered dermal lymphocytes in (**A**). Tissue sample of the vehicle-treated UVA-exposed mice shows the atrophic epidermal changes with keratin plugs (arrows) and degenerated dermal collagen fibers (small arrow) in (**B**). Exposed ectactic dermal blood vessels (small arrow) and dense dermal lymphocytic infiltration (arrow) are shown in (**C**). **D** represents the skin of a metformin 1%-treated mouse showing average thickness of the epidermis with mild dermal lymphocytic infiltration (arrow) and degenerated vacuolated dermal collagen (small arrow). In **E**, almost normal epidermis with minimal dermal lymphocytic infiltration (arrow) and restored dermal collagen are observed in section form metformin 10%-treated mouse. The oral metformin treatment group is represented in **F** by moderate dermal lymphocytic infiltration (arrow) and slight homogenization of the dermal collagen (small arrow). **G** represents the coQ10-treated group and shows mild lymphocytic infiltration (arrow) with restored dermal collagen (small arrow) with similar findings observed by the combined treatment as shown in (**H**). The morphometric analysis of the detected histopathological changes in the form of the epidermal atrophic changes, dermal connective tissue, inflammatory and telangiectatic changes. as well as the overall composite skin changes are presented in (**I-M**), by scatter plots of individual values and medians (transverse lines). * *P* < 0.05 versus normal control; # *P* < 0.05 versus veh-treated UVA group; § *P* < 0.05 versus Met 10%-UVA group, **†** *P* < 0.05 versus Met oral-UVA group. UVA ultraviolet A irradiation, Veh vehicle, Met metformin, CoQ10 coenzyme Q10

Microscopic examination of the skin of vehicle-UVA-exposed mice revealed different signs of photoaging in the form of atrophic epidermis with dilated pilo-sebaceous glands filled with keratin plugs. The dermis showed telangiectasia with perivascular lymphocytic infiltration and a grenz zone of degenerated elastic fibres with amorphous fibrillary material of degenerated elastic fibres. Variable degrees of improvement of the different signs of photoaging were detected in drug treated mice. (Fig. 4A-H) Statistical morphometric analysis of the composite scores of these epidermal and dermal changes showed significant improvement by treatment with metformin 10%, CoQ10, and the combined treatment, while non-significant changes were observed in the metformin 1% or with oral metformin versus the vehicle-UVA-exposed mice. (Fig. 4I-M).

### Effect of drug treatment on autophagic changes associated with UVA exposure

Changes in autophagic machinery were interpreted by the immunostaining results of LC3-II, the most widely used marker for autophagosome formation in conjunction with P62, the specific cargo receptor, whose accumulation is a marker of autophagy blockade distal to autophagosome formation.

### Changes in LC3-II immunostaining

**Fig. 5:**
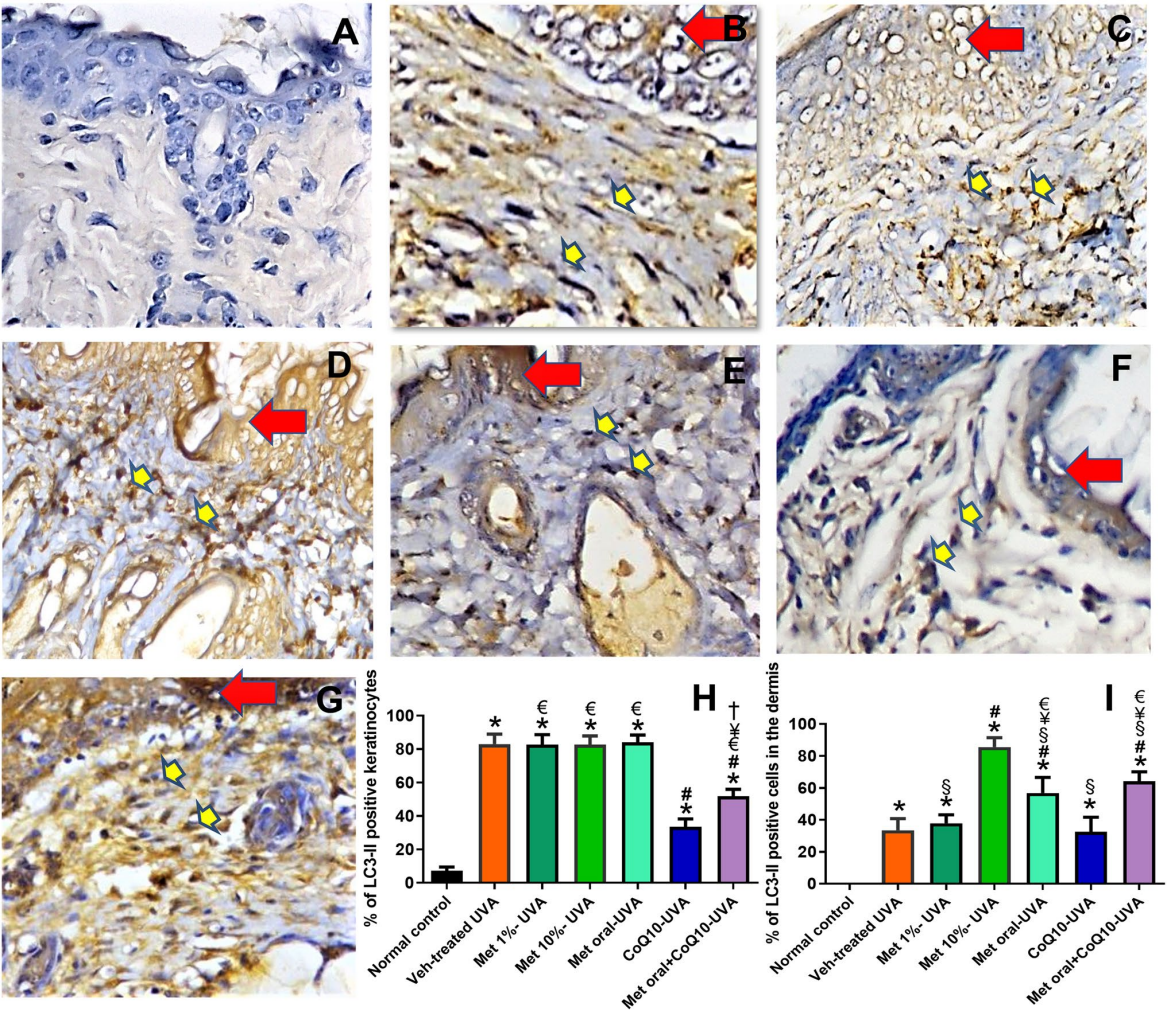
Immunohistochemical staining of LC3 antibody (Mic. Mag. × 200). Representative photomicrographs of immunohistochemical stained skin tissue sections showing negative staining of normal skin in (**A**). Intense staining of the keratinocytes (red arrows) while moderate staining of the dermal fibroblasts and macrophages (yellow arrows) are shown in (**B**), which represents the Veh-treated UVA group. Drug treatment variably affected the number of LC3-stained cells in the epidermis and dermis represented from (**C-G**), as Met 1%, Met 10%, Met oral, CoQ10, and combined Met oral + CoQ10, respectively. Data obtained by software image analysis of the immunohistochemical stained skin sections are represented in (**H** and **I**); as mean ± SD of the percentage positive epidermal and dermal cells, respectively. * *P* < 0.05 versus normal control, **#** *P* < 0.05 versus veh-treated UVA group, § *P* < 0.05 versus Met 10%-UVA group, **†** *P* < 0.05 versus Met oral-UVA group € *P* < 0.05 versus CoQ10-treated UVA, ¥ *P* < 0.05 versus Met 1%-treated UVA. UVA ultraviolet A irradiation, Veh vehicle, Met metformin, CoQ10 coenzyme Q10

Exposure to UVA was associated with a significant increase in the percentage of LC3-II positive cells shown as dot-like staining in the dermal fibroblasts and infiltrating inflammatory cells versus the skin of the normal control, where LC3-II positive cells were either scanty or undetectable in the epidermal and dermal cells, respectively. A further increase in the percentage of LC3-II positive cells was significantly observed in the dermal cells of the metformin 10% treated mice and to a lesser extent in the oral metformin and the combined treatment mice versus vehicle-treated UVA exposed mice. Notably, no significant changes were observed in the dermis of the CoQ10 or the 1% metformin-treated mice. In the epidermis, a marked increase in the percentage of LC3-II positive keratinocytes was significantly observed in the UVA-exposed mice, that was only significantly partially inhibited in the coQ10-treated groups versus the vehicle-treated UVA-exposed mice. (Fig. 5).

### Changes in P62 immunostaining

**Fig. 6:**
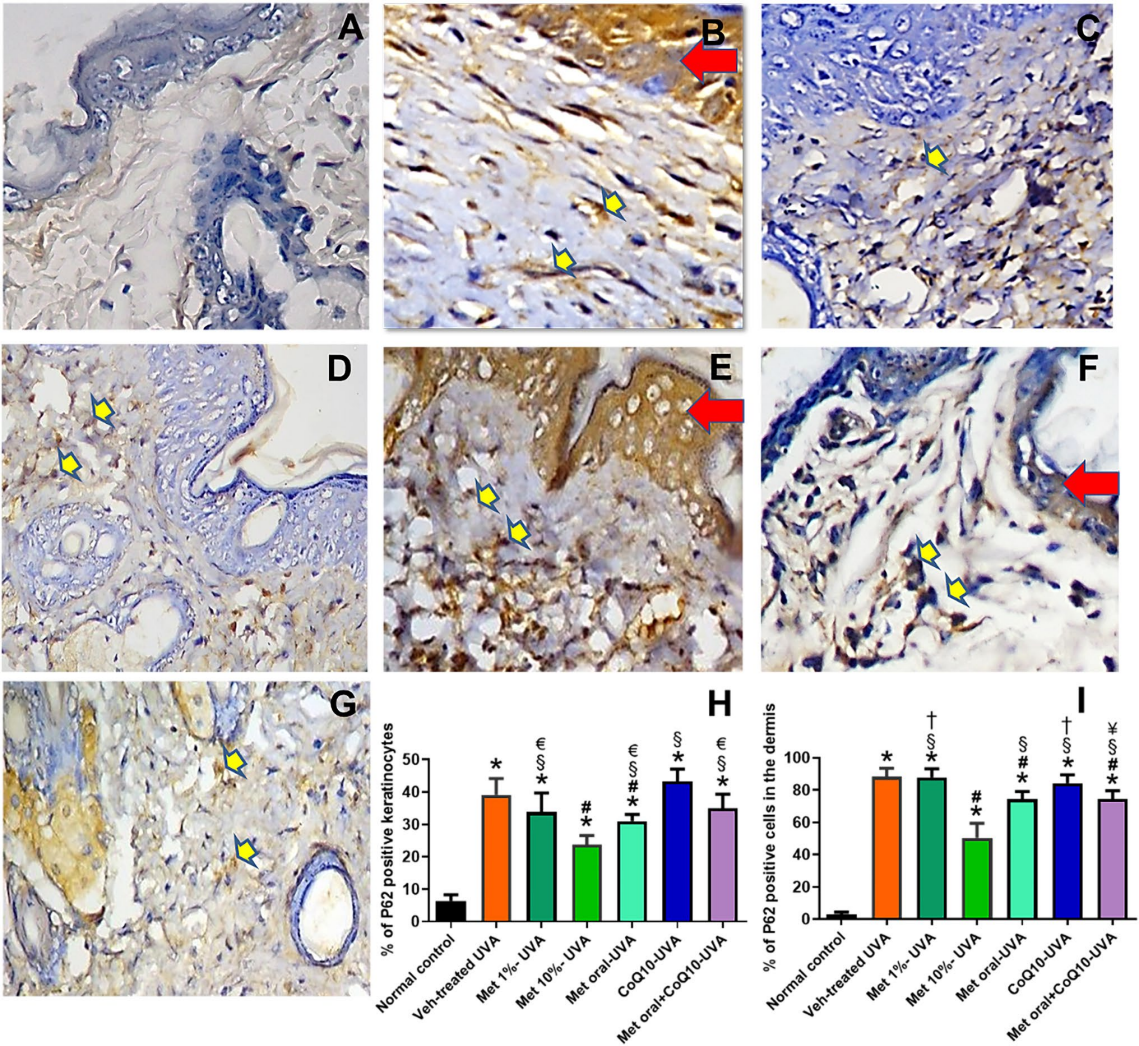
Immunohistochemical staining of P62 antibody (Mic. Mag. × 200). Representative photomicrographs of immunohistochemical stained skin tissue sections showing faint negligible staining in the epidermis with scattered positive dermal fibroblasts and macrophages in normal skin in (**A**). Moderate staining of the keratinocytes (red arrows) while intense staining of the dermal fibroblasts and macrophages (yellow arrows) are shown in (**B**), which represents the Veh-treated UVA group. Variable degrees of reduction of number of P62-stained cells are shown in the skin of the different drug-treated groups represented from (**C-G**); as Met 1%, Met 10%, Met oral, CoQ10, and combined Met oral + CoQ10, respectively. Data obtained by software image analysis of the immunohistochemical stained skin sections are represented in (**H**&**I**); as mean ± SD of the percentage positive epidermal and dermal cells, respectively. * *P* < 0.05 versus normal control, **#** *P* < 0.05 versus veh-treated UVA group, § *P* < 0.05 versus Met 10%-UVA group, **†** *P* < 0.05 versus Met oral-UVA group, € *P* < 0.05 versus CoQ10-treated UVA, ¥ *P* < 0.05 versus Met 1%-treated UVA. UVA ultraviolet A irradiation, Veh vehicle, Met metformin, CoQ10 coenzyme Q10

A significant increase in the percentage of P62 positive cells was observed in all UVA-exposed mice versus the normal control, which showed scanty epidermal and dermal cellular staining. Neither coQ10 nor metformin 1% significantly changed dermal P62 staining, while a significant decrease was shown in metformin 10%, oral metformin, and the combined treated group. (Fig. 6) A similar trend was observed in the keratinocytes, but the significant decrease in P62 staining was detected only with the oral and 10% metformin.

### Effect of drug treatment on apoptotic changes associated with UVA exposure

**Fig. 7:**
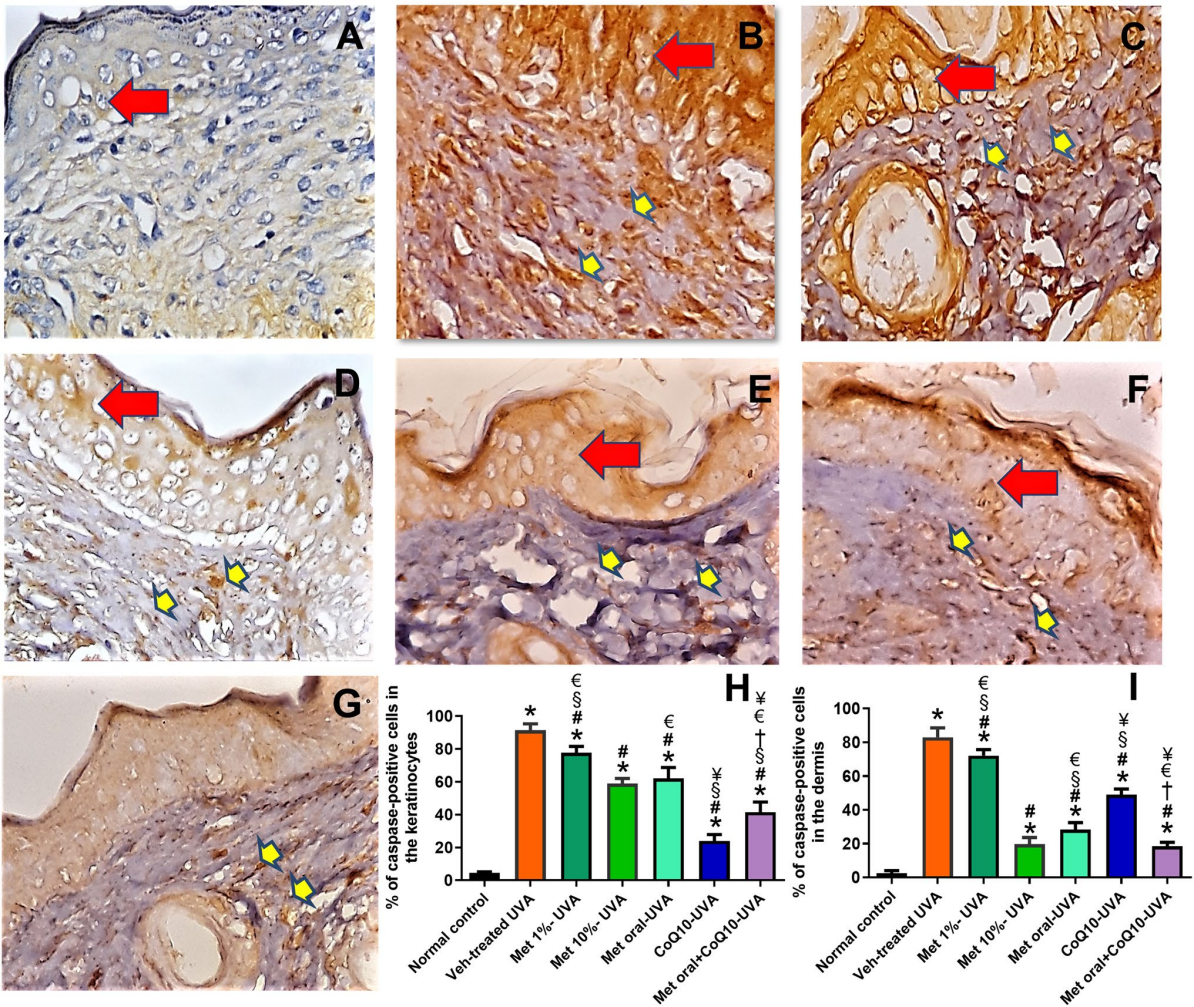
Immunohistochemical staining of anti-caspase antibody. (Mic. Mag. × 200). Representative photomicrographs of immunohistochemical stained skin tissue sections showing scattered positive cell of normal skin in (**A**). Intense staining of the keratinocytes (red arrows) as well as the dermal fibroblasts and macrophages (yellow arrows) are shown in (**B**), which represents the Veh-treated UVA group. Drug treatment reduced the number of anti-caspase-stained cells in the epidermis and dermis represented from (**C-G**), as Met 1%, Met 10%, Met oral, CoQ10, and combined Met oral + CoQ10, respectively. Data obtained by software image analysis of the immunohistochemical stained skin sections are represented in (**H**&**I**), as mean ± SD of the percentage positive epidermal and dermal cells, respectively. * *P* < 0.05 versus normal control, **#** *P* < 0.05 versus veh-treated UVA group, § *P* < 0.05 versus Met 10%-UVA group, † *P* < 0.05 versus Met oral-UVA group, € *P* < 0.05 versus CoQ10-treated UVA, ¥ *P* < 0.05 versus Met 1%-treated UVA. UVA ultraviolet A irradiation, Veh vehicle, Met metformin, CoQ10 coenzyme Q10

Skin specimens of all UVA-exposed mice showed a significant increased number of caspase 3 positive cells in the epidermis and dermis versus the normal control mice. Drug treatment significantly decreased the expression of caspase 3 with the least observed in the metformin 1%-treated mice. (Fig. 7).

## Discussion

Autophagy is a major counter-regulatory process that supports skin, not only to maintain homeostasis, but also to face environmental stresses (Eckhart et al. 2019. We investigated the benefit of using topical and oral metformin, as a strong autophagy inducer in photoaging, alone and combined with CoQ10. Consistent with previous studies, UVA-exposure was associated with increased skin oxidative stress, overwhelming the endogenous antioxidant capacity (increased MDA and decreased GSH). The detected reduction of Nrf-2, the master regulator of antioxidant genes, augmented the oxidative damage. ROS increase the cellular membrane phosphorylation, thereby activate signaling pathway leading to increased MMP-1, MMP-3 secretion, and collagen degradation (Karin 1995; Kim et al. 2018). Indeed, we detected a marked increase in MMP-1 after UVA-exposure, reflected clinically as an elevated photoaging macroscopic score and markedly prolonged pinch test time in the vehicle-treated UVA-exposed mice. Likewise, collagen fragmentation was revealed microscopically with dermal inflammatory cell infiltration. In this context, excess ROS was documented to upregulate nuclear factor kappa β (NF-κβ), leading to IL-6 production as demonstrated herein. This can further induce cyclooxygenase-2 and prostaglandin production, causing inflammation (Ravi and Piva 2013).

On tracing autophagy, a significantly increased LC3-II and P62 in the dermal cells was retrieved. Though the increase in LC3-II may signify autophagy induction, the associated marked increase in P62 denotes that LC3-II accumulation is rather a result of distal autophagy blockade beyond autophagosome formation; probably at the lysosomal level (Schläfli et al. 2016; Schmitz et al. 2016). In line, an inhibitory effect of UVA on dermal fibroblast autophagic flux through a unique mechanism involving lysosomal inhibition was demonstrated (Huang et al. 2019. We detected a remarkably reduced cathepsin D in the UVA-exposed mice, a finding that explains the observed autophagy blockade and the enhanced apoptosis. In line, Hah et al. (2012) suggested that stress-induced cell death can be suppressed by activating cathepsin D-dependent autophagy. Indeed, the impact of dysregulated autophagy is usually more pronounced in the long-lived differentiated cells and thus changes in the dermal fibroblasts are considered the main contributors to photoaging by activating senescence or apoptosis, which is context dependent (Eckhart et al. 2019). This is due to the deep penetration of the UVA into the dermis. The UVA induced-ROS inflict indirect oxidative damage rather than the direct UVB-induced DNA damage (De Gruijl et al. 2002). Loss of the capacity to remove oxidized and misfolded proteins due to autophagic blockade can activate DNA repair pathways and thereby inducing cellular senescence (Sample and He 2017; Eckhart et al. 2019).

Alternatively, ROS modulate apoptotic signals by downregulating the survival signal Bcl-2 and upregulating the death factor Bax (Lu et al. 2004). In line, we demonstrated an increased anti-caspase 3 dermal immunostaining in the UVA-exposed mice supporting that autophagic blockade led to enhanced apoptosis. Moreover, defective autophagy triggers severe inflammation in the skin, because of inflammasome activation with the aberrant liberation of proinflammatory cytokines (Wang et al. 2019); as reflected by the observed increase in IL-6 with subsequent dermal lymphocyte and macrophage infiltration.

Our findings displayed that improving the autophagic flux by drug treatment alleviated the UVA-induced photoaging. Though the topical application of metformin 1% was not associated with a significant amelioration of the micro/macro-scopic signs of photoaging, a marked improvement was observed with the 10% metformin, pointing to a probable dose-dependent effect. Oral metformin administration was associated with significant improvement but to a lesser extent.

The reported metformin inhibitory effect on MMPs with upregulation of collagen-1 production by Cui et al. (2019), goes well with the observed normalization of MMP-1 and the restoration of dermal collagen demonstrated herein with metformin 10%. Metformin also reduced the inflammatory dermal infiltrate endorsed by decreasing IL-6. In line, metformin inhibited inflammatory responses by suppressing NF-κβ signalling and subsequently mitigating proinflammatory cytokine production (Ba et al. 2019).

The favourable metformin effects on redox balance and antioxidant gene expression, manifested here by increased GSH coupled with a decreased MDA, can further explain its remarkable improvement on photoaging. In concordance, Cui et al. (2019) demonstrated decreased ROS by metformin in UVA-irradiated human skin fibroblasts. Activation of AMPK with inhibition of mitochondrial respiratory chain have long been recognized as the primary mechanisms of metformin actions (Novelle et al. 2016). Combined with the currently observed inducing effect of metformin on Nrf-2 expression, these actions can provide mechanistic insights into metformin’s role in lowering ROS and their induced cellular damage.

Nevertheless, the observed metformin effect on autophagy may represent the centrepiece harmonizing all these actions. Notably, the autophagy-lysosomal pathway is particularly unique in degrading redox damaged proteins without aberrantly changing the redox network needed for metabolism or signalling (Dodson et al. 2015). We demonstrated an increased number of LC3-II positive cells in the dermis, which is consistent with the known autophagy inducing effect of metformin through AMPK activation. Of note, activating autophagy in the presence of the downstream blockade induced by UVA was expected to result into more accumulation of P62. Conversely, we observed a decrease in P62 versus photoaged mice denoting a relief of autophagic blockade rather than the mere activation of proximal autophagic events. In fact, evidence on metformin role on late phase of autophagy is just being uncovered. Kanamori et al. (2019) demonstrated metformin cardioprotective effects by enhancing autophagic flux, via upregulation of cathepsin D. Indeed, this is of special importance considering our findings that cathepsin D expression was markedly reduced by UVA exposure and this reduction was reversed by topical 10% metformin. The notion that pharmacological cathepsin D inactivation mimics UVA-induced autophagic dysfunction in human dermal fibroblasts further supports our data (Lamore et al. 2010; Lamore and Wondrak 2012). The enhancement of autophagic flux by metformin was further reflected by decreased apoptotic changes and declined signs of photoaging.

The topical 10% metformin was probably better than oral metformin due to higher local concentration in the skin. Of interest, metformin transdermal delivery has recently gained attention (Ng and Gupta 2020). Studies for repurposing metformin for dermatological disorders by topical applications are ongoing, as well (Polonini et al. 2019).

While the dermal resident cells, particularly the fibroblasts, are mostly affected by defective autophagy, the condition may be different with short-lived differentiated keratinocytes (Eckhart et al. 2019). These cells have high basic autophagy to maintain intracellular homeostasis. Because of their fast turnover, their autophagic activity are less liable to be impaired with external and internal factors. Conversely, they have a vast capacity to respond to stress by increasing their basic autophagic activity leading to hyperkeratosis and epidermal hyperplasia, which protect against the UV ray-penetration to the skin.

In epidermal cells, we observed a marked increase in LC3-II without a comparable accumulation of P62 after UVA exposure, consistent with activated autophagy. Though autophagy might be one of the cytoprotective mechanisms against the UV-induced apoptosis in keratinocytes (Misovic et al. 2013; Li et al. 2016), when excessive, it turns maladaptive and may trigger apoptosis. In canonical autophagy pathway, a possible switch controlling autophagy and apoptosis in keratinocytes was previously described (Maiuri et al. 2007). This explains the marked increase in caspase 3 positive keratinocytes with epidermal atrophy rather than hyperplasia. Therefore, the keratinocytes overstimulated autophagy encountered here seems to be detrimental.

Considering the mutual interplay between autophagy and oxidative stress, the introduction of antioxidants in combatting UVA-hazard may be of a special benefit. We explored the additive effect of the effective antioxidant coQ10. CoQ10-treatment markedly reduced MDA in association with a subtle but non-significantly low GSH level. This is explained by the novel concept of mitohormesis, where supplementation of agents with direct antioxidant activity may limit local endogenous antioxidant production (Ristow et al. 2009). CoQ10 acts as a diffusible electron carrier in the mitochondrial respiratory chain quenching free radicals and inhibiting the initiation and propagation of lipid peroxidation in cellular bio-membranes (Luo et al. 2019). The reduced oxidative stress, itself, may decrease the excess autophagy activation as depicted from our findings and therefore was associated with a better antiapoptotic effect in the keratinocytes, both in the coQ10 and the combined treatment group. In line, coQ10 was reported to have antiapoptotic and anti-inflammatory activities (Yousef et al. 2019). However, this effect was less observed on dermal cells due to the more destructive effect of deeply infiltrating UVA on dermal structures than keratinocytes (Muta‐Takada et al. 2009). Also, the great localization of CoQ10 in the stratum corneum supports this assumption (Flora 2009). Nevertheless, the relative restoration of collagen fibres may be related to its modest effect on reducing the UVA-induced MMP-1, as shown by our data. The activating effect of CoQ10 on the synthesis of collagen type IV and VII, responsible for the integrity of the skin basement membrane and epidermis, and the stimulatory effect on elastin gene expression in dermal fibroblasts may also provide another explanation (Zhang et al. 2012).

## Conclusion

Autophagy meticulous control plays a critical role in skin defences against UVA-induced photoaging to avoid overriding responses. Through modulation of autophagy, metformin, mainly topically, improved clinical and histologic signs of photoaging. Metformin effects may not only be related to induction of autophagy but relief of autophagy blockade, through cathepsin induction, is crucial. Though the oral metformin had much less effect than its topical form, the co-application of coQ10 boosted its effects where each of them compensated for the defective action of the other. Overall, these results pave the way for the potential use of metformin in photoaging.

## Data Availability

The datasets generated during and/or analysed during the current study are available from the corresponding author on reasonable request.

## Abbreviations

UVA: Ultraviolet A
MED: Minimal erythema dose
coQ10: Coenzyme Q10
MDA: Malondialdehyde
IL-6: Interleukin 6
GSH: Reduced glutathione
ROS: Reactive oxygen species
MMP-1: Matrix metalloproteinase-1
LC3-II: Light chain 3 alpha II
Nrf-2: Nuclear factor erythroid-related factor 2
BSA: Bovine serum albumin
TBST: Tris-buffered saline containing Tween 20
